# Detection and characterization of zoonotic dermatophytes from dogs and cats in and around Kolkata

**DOI:** 10.14202/vetworld.2015.1078-1082

**Published:** 2015-09-19

**Authors:** S. Murmu, C. Debnath, A. K. Pramanik, T. Mitra, S. Jana, S. Dey, S. Banerjee, K. Batabyal

**Affiliations:** 1 Department of Veterinary Public Health, Faculty of Veterinary and Animal Sciences, West Bengal University of Animal and Fishery Sciences, Kolkata - 700 037, West Bengal, India; 2 Swastha Bhavan, Ministry of Health & Family Welfare, Government of West Bengal, GN 29, Salt lake, Sector V, Kolkata, West Bengal, India; 3 Department of Veterinary Microbiology, Faculty of Veterinary and Animal Sciences, West Bengal University of Animal and Fishery Sciences, Kolkata - 700 037, West Bengal, India

**Keywords:** cats, dermatophytes, dogs, humans, zoonotic infections

## Abstract

**Aim:**

The ringworms of pet dogs, cats, and stray animals (dogs, cats, and other animals) could be a potential source of zoonotic infections causing a serious public health problem in the busy city Kolkata. The pet owners are more susceptible to get this infection from their pets, because of the close contact with them as dermatophytosis is very much prevalent in those pets. So, this study was aimed to check the prevalence of dermatophytosis in dogs, cats, and in pet owners.

**Materials and Methods:**

A total of 362 clinically suspected cases of dermatophytosis from dogs (123 in number), cats (202 in number), and human beings (37 in number) were collected and studied from in and around Kolkata to detect the presence of significant dermatophytes. Direct microscopy and cultural examination of the isolates were performed following standard methodology. Identification and characterization of the isolates were done by different biochemical tests.

**Results:**

Samples (n=285) having significant dermatophytic fungal infections were found to be of highest number in cats (158, 55.5%) than in dogs (108, 37.8%) and humans (19, 6.7%), respectively. The incidence of *Microsporum canis* (60.0%) was the highest from affecting dogs, cats, and human beings in comparison to *Microsporum gypseum* (22.5%), *Trichophyton mentagrophytes* (15.8%) and *Trichophyton rubrum* (1.7%). Detection of *T. rubrum* was only from human cases in this study, whereas the presence of rest three were slightly higher in cats than that of the dogs and humans in this present study. The incidences were higher in young animals and in humans of the age group of 21-30 years, during the rainy season (from April to August) and also in in-contact human beings.

**Conclusion:**

*M. canis* was the most commonly pathogen among all causing dermatophytosis in animals and also in the pet owners. *M. gypseum* and *T. mentagrophytes* were other pathogens associated with these infections. These infections were more prevalent in the rainy seasons and in in-contact human patients or pet owners.

## Introduction

Dermatophytosis, an integumentary, cosmopolitan mycotic disease, is important from public health as well as economic point of view and is prevalent both in sporadic and epidemic forms over 145 countries of the world including India. It is an important occupational mycozoonoses of dairymen, animal handlers, livestock farmers, pet owners, veterinarians, etc. [1]. The prevalence of superficial mycoses caused by zoophilic dermatophytes were found to be significantly positive in different parts of the world [2] and in the tropical country with warm and humid climate, crowded living and poor sanitary conditions like India [3]. It is noticed that almost 20-50% human skin infections were from zoonotic dermatophytes [3,4] mainly found in pet animals which can be transmitted to other animals also (*Microsporum canis* and *Trychophyton mentagrophytes*) [4]. Authors such as Day *et al*. [5] and Moretti *et al*. [6] reported the prevalence of more than 50-70% cases human mycotic infections from animal hosts or mainly the pet animals. Poor management of pets can increase the no. of infected pets [5] irrespective of their age followed by infection in humans where they colonize in the keratinized outermost layer of the skin with significant lesions [6]. Among the pet animal cats and dogs may be the most susceptible to the disease and in urbanized cities like Kolkata, they might be the main source of human fungal infections which are very difficult to treat [7]. The prevalence and distribution of the dermatophytoses in those pet animals and pet owners in the light of its zoonotic potentiality in and around Kolkata city, West Bengal, India is hereby studied considering the above background.

## Materials and Methods

### Ethical approval

The study was approved by Institutional Biosafety Committee, West Bengal University of Animal and Fishery Sciences, Kolkata and as per the Committee for the Purpose of Control and Supervision on Experiments on Animals (CPCSEA) rules; it does not require any approval of Institutional Animal Ethics Committee. Dr. S Jana, Medical Officer, Swastha Bhavan, Ministry of Health & Family Welfare, Govt. of W.B. helped the researchers in collection of samples from the Pet Owners with proper permission.

### Collection of samples

The samples such as skin, hair, claw, hoof, and nails were collected from infected pet animals (dogs and cats) with dermatophytosis and human beings from Kolkata and its’ adjacent area during the period from January to August, 2013. A total of 362 cases were examined to collect samples from cats (202 in number), dogs (123 in number), and human being (37 in number) with the evidence of dermatophytosis such as hair loss, scaling, crusts, and desquamation. Age- and season-wise categorization of samples was done for future study.

### Direct microscopic examination

The suspected materials were placed in a drop of 10% aqueous solutions of potassium hydroxide (KOH) on a clean glass slide was added to the KOH solution with or without colorant, to facilitate demonstration of the fungal elements. A glass cover slip was placed a top of the preparation and the slide was gently warmed over a flame, avoiding boiling. The slide was then allowed to cool for a few minutes and was blotted gently to remove excessive KOH solution for the better observation of fungal hyphae, macroconidia, arthrospores, etc.

### Cultural examination

All collected fungal samples were considered for cultural examination followed by characterization according to their colony characteristics, conidial cell structure, the size shape and presence of septae with number and arrangement of conidial cells around the hyphae [7]. The medium, i.e., Sabouraud’s dextrose agar with 0.05% chloramphenicol, containing chloramphenicol and cycloheximide was more suitable for primary isolation since they suppressed bacteria and saprophytic fungal growth respectively.

### Inoculation of medium

Hair fragments, skin scrapings, specimens from vesicles and blisters, nails, and hooves were inoculated after primary seeding in three sets of test tubes - one containing Sabouraud’s dextrose agar with 0.05% chloramphenicol, second containing Sabouraud’s dextrose agar with 0.05% chloramphenicol plus 0.05% cycloheximide and third onto dermatophyte test medium followed by incubation at 28°C for up to 4 weeks for the first two sets and for up to 10 days for the last set with periodical examination for any growth/color change.

### Identification

Fungal isolates were identified following “dermatophyte identification scheme” by Koneman and Roberts [8] with macroscopical examination of cultures including study of colony morphology, pigmentation, growth rate and microscopic examinations by lactophenol cotton blue staining and slide culture technique (Riddell’s method). Other tests such as urease test, *in-vitro* hair perforation test, temperature tolerance, rice grain test, growth pattern on trichophyton agar, and corn meal agar test (for pigmentation if any) [4] were also carried out to confirm the presence of different isolates.

### Statistical analysis

Data obtained in this study were analyzed by statistical methods using General Linear Model of IBM SPSS software package, version 20, developed as per the procedure of Snedecor and Cochran [9].

## Results

**Figure-1:**
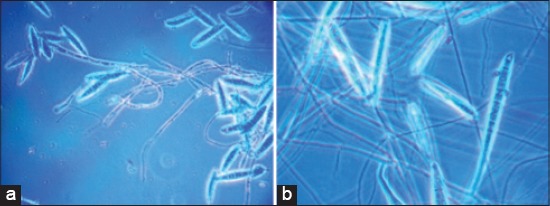
(a and b) *Microsporum canis* and *Microsporum gypseum* with well-developed macroconidia and stalked microconidia.

After isolation and thorough characterization, a total of 285 (78.7%) samples were found to be positively infected with different dermatophytes among that prevalence of infection was the highest in cats (158, 55.5%) than dogs (108, 37.8%) and human beings (19, 6.7%). The colonies of *Microsporum canis* and *Microsporum gypseum* were wooly aerial mycelium, light to reddish brown pigmentation. The microscopical study revealed the presence of well-developed macroconidia with 6-12 septa and small microconidia with stalked appearance (Figure-1a and b). The isolates of *Microsporum* spp. showed luxuriant growth on rice grain medium with reddish to orange pigmentation.

**Figure-2:**
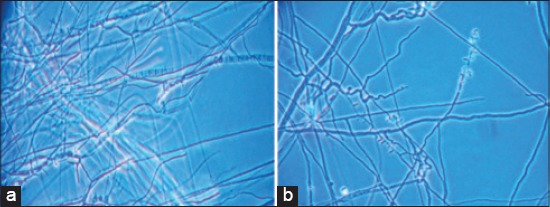
(a and b) *Trychophyton mentagrophytes* and *Trychophyton rubrum* showing micro and macroconidia with chlamydospores.

Characterization of *T. mentagrophytes* and *Trichophyton rubrum* were done on the basis of their smooth cottony colonies with white to yellowish pigmentation. Under the microscope, there were both micro (more in number) and macroconidia with chlamydospores (Figure-2a and b). Isolates of *T. mentagrophytes* were positive to urease (in 5-7 days) and hair perforation tests whereas *T. rubrum* isolates were negative. *T. mentagrophytes* species grew slowly at 37°C and luxuriantly on Trichophyton agar medium No. 1 and 4 with whitish colonies whereas *T. rubrum* could not grow at 37°C and showed huge bright red color growth on trichophyton agar medium No. 1 and 4. Growth on corn-meal dextrose agar of *T. mentagrophytes* was consistently yellow pigmented in comparison to the reddish growth of *T. rubrum*.

Incidence of *M. canis* was the highest (60%) among all infected animals and human patients in comparison to *M. gypseum* (22.5%), *T. mentagrophytes* (15.8%), and *T. rubrum* (1.7%) (Table-1). Detection of *M. canis* (61.4%) and *M. gypseum* (22.8%) from cats were the highest than from dogs and human beings. The incidence of *T. mentagrophytes* in dogs (16.7%), and cats (15.8%) were also much higher than that of human beings. Detection of *T. rubrum* was observed only from human cases (26.4%) which are quite significant.

**Table-1:** Prevalence of dermatophytes in different hosts.

Dermatophytes	Cumulative		Dogs		Cats		Humans	
			
	No.	%	No.	%	No.	%	No.	%
*M. canis*	171	60.0	66	61.1	97	61.4	8	42.1
*M. gypseum*	64	22.5	24	22.2	36	22.8	4	21.0
*T. mentagrophytes*	45	15.8	18	16.7	25	15.8	2	10.5
*T. rubrum*	5	1.7	0	0	0	0	5	26.4
Total	285	100.0	108	100.0	158	100.0	19	100.0

T. mentagrophytes=Trychophyton mentagrophytes, M. canis=Microsporum canis, M. gypseum=Microsporum gypseum, T. rubrum=Trychophyton rubrum

Incidence of infection was significantly higher in male dogs (58.3%) in comparison to bitches (41.7%) but in case of cats, difference in incidence between male (51.3%) and female ones (48.7%) were found to very minimum. Male patients (78.9%) showed significantly higher infection rate than their female counterparts (21.1%) (Table-2).

**Table-2:** Sex-wise incidence of dermatophytes in different hosts.

Hosts	Dogs		Cats		Humans	
		
	No. of cases	%	No. of cases	%	No. of cases	%
Positive cases	108		158		19	
Sex						
Male	63	58.3	81	51.3	15	78.9
Female	45	41.7	77	48.7	04	21.1

Incidences of infection were higher in adult dogs (61.1%), adult human patients (57.9%), and in kittens (56.3%) (Table-3). In cases of human patients, patients of the age group of 21-30 years suffered most (36.8%) from fungal infections as detected in this study (Table-4). The human cases were reported to be more common in in-contact patients (16, 84.2%) than that of non-contact ones (3, 15.8%) (Table-5). This study revealed significant seasonal variation of incidence of infection among different hosts with the rainy season (from June to August) to possess the highest incidence rate (74.8%) in comparison to other seasons (Table-6).

**Table-3:** Age-wise incidence of dermatophytes in animals and humans.

Hosts	Dogs		Cats		Humans	
Positive cases	108		158		19	
Age	Adults	Puppies	Adults	Kitten	Adults	Children
No. of cases	66	42	69	89	11	08
%	61.1	38.9	43.7	56.3	57.9	42.1

**Table-4:** Age group distribution within the human patients.

Age (years)	No. of positive cases	%	Age (years)	No. of positive cases	%
≤10	02	10.5	4150	01	5.3
1120	06	31.6	5160	01	5.3
2130	07	36.8*	6170	00	0.0
3140	02	10.5	Total:	19	100.0

* (p>0.05)

**Table-5:** Isolation rates of dermatophytes from in-contact human patients.

Area	In-contact patients		Non-contact patients	
	
	No. of +ve cases	Isolation (%)	No. of +ve cases	Isolation (%)
Rural	07	36.8	01	5.3
Urban	09	47.4**	02	10.5
Total	16	84.2	03	15.8

** (p>0.05)

**Table-6:** Seasonal distribution of dermatophytes.

Seasons	Months	Distribution of dermatophytes				Total	
	
		Dogs and cats		Humans		No.	%
	
		No.	%	No.	%		
Winter	January	3	1.1	0	0	11	3.8
	February	8	2.8	0	0		
Summer	March	6	2.1	0	0	61	21.4
	April	10	3.5	2	0.7		
	May	41	14.4	2	0.7		
Rainy season	June	67	23.5	6	2.1	213	74.8
	July	79	27.7*	7	2.5**		
	August	52	18.2	2	0.7		
Total		266	93.3	19	6.7	285	100.0

* (p<0.05)

** (p=0.025)

## Discussion

Prevalence of dermatophytic infections in cats was the highest (55.5%) in than dogs and human beings. This high rate of prevalence in cats was also supported by Nweze [10], and Esch and Peterson [11] who observed 58-67% occurrence rate in their studies. The prevalence of infections in dogs is in accordance with the reports of Brilhante *et al*. [12] and Seker and Dogan [13] (25-45% prevalence rate of fungal infections in dogs). Authors such as Kasai [14] and Falahati *et al*. [15] reported slightly higher prevalence rate (13-14%) of fungal infections in humans from pets but Stojanov *et al*. [16] reported 5-8% positive human cases which are in line with this report (6.7% positivity).

Characterization of isolates of *Microsporum canis*, *M. gypseum*, *Trychophyton mentagrophytes*, and *T. rubrum* were performed as per “dermatophyte identification scheme” [10] which are in accordance with the studies of Brilhante *et al*. [12], Seker and Dogan [13], and Falahati *et al*. [15].

The most common dermatophyte reported to infect animals as well as human beings was *M. canis* (60%) [15,17]. Brilhante *et al*. [12] and Seker and Dogan [13] also placed *M. canis* at top in order of prevalence followed by *M. gypseum* and *T. mentagrophytes* in pet animals such as dogs and cats which are in line with the findings of this study. Human patients were mostly infected with all these zoonotic pathogens with *M. canis* to be the most prevalent one followed by *T. rubrum*, *M. gypseum*, and *T. mentagrophytes* which were also seen by Kasai [14], Falahati *et al*. [15] and Venkatesan *et al*. [18].

The present study revealed that the prevalence of fungal infection were in male dogs which are in agreement with the reports of Falahati *et al*. [15], Brilhante *et al*. [12], and Seker and Dogan [13] who reported more infection rate in male dogs (19-20%) than bitches (16-17%). However, they [12,13,15] found no significant differences between male and female cats in incidence of fungal skin infections in their studies but Alpun and Ozgur [19] reported a slightly higher incidence of dermatophytoses in male cats which are in line with this study.

Male patients were mostly affected than the female ones which are in agreement with the reports of Falahati *et al*. [15] (65.7% in males and 34.4% in females), Ngwogu and Otokunefor [20] (29% in males and 1.4% in females), which might be due to the more association of males with the suffering animals or pets. Adult dogs, adult human patients and kittens were found to be more prone to infection in this study which may be due to more association of these owners with their pets such as adult dogs and kittens (very lovable pets which generally like to stay in the lap of the owners), thus the infection rate is lower in adult cats but the difference is not very much significant. These findings are in accordance with the works of Brilhante *et al*. [12] and Seker and Dogan [13]. Findings of Falahati *et al*. [15] and Gangil *et al*. [21] are also in full agreement with the age group distribution of human isolates as noticed in this study that the adult human owners (of 21-30 years of age) were mostly affected as they remain more closely attached with their pets (whether infected or healthy) than the older ones or younger ones, resulting in a higher exposure to infection also. Authors such as Maraki *et al*. [22], Falahati *et al*. [15], and Seker and Dogan [13] also reported the higher prevalence of infection in in-contact human patients and in urban area as also reported in this study. Significantly higher rate of infection in the rainy season in comparison to other seasons are quite relevant as this matches with the findings of Falahati *et al*. [15] and Maraki *et al*. [22].

## Conclusion

Therefore, the present study revealed that fungal infections are mostly prevalent during rainy seasons, in pet animals such as cats and dogs which can act as potential sources for human infections mainly in in-contact human beings. Again *M. canis* is the major pathogen causing infections in susceptible hosts of different ages. So, proper care should be taken during management of pet animals to minimize human infections.

## Authors’ Contributions

SM, TM, SB, and CD chalk out the study design and carried out the experiment. SM with SJ collected the human samples for this study. AKP, KB, and SD analyzed the data, drafted and revised the manuscript. All authors read and approved the manuscript.

## References

[1] Ruben L.M. (2010). Candidosis, a new challenge. Clin. Dermatol.

[2] Akpolat N.O., Akdeniz S., Elci S., Atmaca S., Ozekinci T. (2005). Tinea capitis in Diyarbakir, Turkey. Mycoses.

[3] Weese J.S., Fulford M. (2010). Companion Animal Zoonoses.

[4] Scott D.W., Miller W.H., Griffin C.E. (2001). Fungal skin disease. Muller and Krik's Small Animal Dermatology.

[5] Day M.J., Breitschwerdt E., Cleaveland S., Karkare U., Khanna C., Kirpensteijn J., Kuiken T., Lappin M.R., McQuiston J., Mumford E., Myers T., Palatnik-de-Sousa C.B., Rubin C., Takashima G., Thiermann A. (2012). Surveillance of zoonotic infectious disease transmitted by small companion animals. Emerg. Infect. Dis.

[6] Moretti A., Agnetti F., Mancianti F., Nardoni S., Righi C., Moretta I., Morganti G., Papini M. (2013). Dermatophytosis in animals: Epidemiological, clinical and zoonotic aspects. G. Ital. Dermatol. Venerol.

[7] Nilce M., Martinez R., Nulu T.A. (2008). Antifungal resistance mechanism in dermatophytes. Mycopathologia.

[8] Koneman E.W., Roberts G.D. (1985). Dermatophyte identification schema. Practical Laboratory Mycology.

[9] Snedecor G.W., Cochran W.G. (1994). Statistical Methods.

[10] Nweze E.I. (2011). Dermatophytoses in domesticated animals. Rev. Inst. Med. Trop.

[11] Esch K.J., Peterson C.A. (2013). Transmission and epidemiology of zoonotic protozoal diseases of companion animals. Clin. Microbiol. Rev.

[12] Brilhante R.S.N., Cavalcante C.S.P., Soares F.A., Cordeiro R.A., Sidrim J.J.C., Rocha M.F.G. (2003). High rate of *Microsporum canis* feline and canine dermatophytoses in North-East Brazil: Epidemiological and diagnostic features. Mycopathologia.

[13] Seker E., Dogan N. (2011). Isolation of dermatophytes from dogs and cats with suspected dermatophytosis in Western Turkey. Prev. Vet. Med.

[14] Kasai T. (2000). Epidemiological survey of dermatophytoses in Japan. Epidemiological Investigation committee for human mycoses in the Japanese society for medical mycology. Nippon Ishinkin Gakkai Zasshi.

[15] Falahati M., Akhlaghi L., Lari A.R., Alaghehbandan R. (2003). Epidemiology of dermatophytoses in an area south of Tehran, Iran. Mycopathologia.

[16] Stojanov I.M., Prodanov J.Z., Pusic I.M., Ratajac R.D. (2009). Dermatomycosis –A potential source of zoonotic infection in cities. Proc. Natl. Sci. Matica Srp. Nov Sad.

[17] Mattei A.S., Beber M.A., Madrid I.M. (2014). Dermatophytosis in small animals. SOJ Microbiol. Infect. Dis.

[18] Venkatesan G., Ranjit Singh A.J.A., Muregesan A.G., Janaki C., Gokul Shankar S. (2007). *Trichophyton rubrum* –the predominant etiological agent in human dermatophytoses in Chennai, India. Afr. J. Microbiol. Res.

[19] Alpun G., Ozgur N.Y. (2009). Mycological examination of *Microsporum canis* infection in suspected dermatophytosis of owned and ownerless cats and its asymptomatic carriage. J. Anim. Vet. Adv.

[20] Ngwogu A.C., Otokunefor T.V. (2007). Epidemiology of dermatophytoses in a rural community in Eastern Nigeria and review of literature from Africa. Mycopathologia.

[21] Gangil R., Dutta P., Tripathi R., Singathia R., Lakhotia R.L. (2012). Incidence of dermatophytosis in canine cases presented at Apollo Veterinary College, Rajashtan, India. Vet. World.

[22] Maraki S. (2012). Epidemiology of dermatophytoses in Crete, Greece between 2004 and 2010. G. Ital. Dermatol. Venereol.

